# Vasopressin and Oxytocin in Control of the Cardiovascular System

**DOI:** 10.2174/1570159X11311020008

**Published:** 2013-03

**Authors:** Nina Japundžić-Žigon

**Affiliations:** Professor of Basic and Clinical Pharmacology and Toxicology, University of Belgrade School of Medicine, Institute of Pharmacology, Clinical Pharmacology and Toxicology, Dr Subotica 1, Belgrade, Republic of Serbia

**Keywords:** Vasopressin, oxytocin, respiration, blood pressure, baro-receptor reflex, stress, hypertension, heart failure.

## Abstract

Vasopressin (VP) and oxytocin (OT) are mainly synthesized in the magnocellular neurons of the paraventricular (PVN) and supraoptic nucleus (SON) of the hypothalamus. Axons from the magnocellular part of the PVN and SON project to neurohypophysis where VP and OT are released in blood to act like hormones. Axons from the parvocellular part of PVN project to extra-hypothalamic brain areas (median eminence, limbic system, brainstem and spinal cord) where VP and OT act like neurotransmitters/modulators. VP and OT act in complementary manner in cardiovascular control, both as hormones and neurotransmitters. While VP conserves water and increases circulating blood volume, OT eliminates sodium. Hyperactivity of VP neurons and quiescence of OT neurons in PVN underlie osmotic adjustment to pregnancy. In most vascular beds VP is a potent vasoconstrictor, more potent than OT, except in the umbilical artery at term. The vasoconstriction by VP and OT is mediated via V1aR. In some vascular beds, i.e. the lungs and the brain, VP and OT produce NO dependent vasodilatation. Peripherally, VP has been found to enhance the sensitivity of the baro-receptor while centrally, VP and OT increase sympathetic outflow, suppresse baro-receptor reflex and enhance respiration. Whilst VP is an important mediator of stress that triggers ACTH release, OT exhibits anti-stress properties. Moreover, VP has been found to contribute considerably to progression of hypertension and heart failure while OT has been found to decrease blood pressure and promote cardiac healing.

## INTRODUCTION

Neurohypophyseal peptides vasopressin (VP) and oxytocin (OT) are mainly synthesized in the supraoptic nucleus (SON) and the paraventricular nucleus (PVN) of the hypothalamus. Neurons of SON and of the magnocellular part of the PVN project to neurohypophysis [1]. From there VP and OT are released in the systemic circulation, where they act like hormones, reach distant targets and participate in the regulation of many functions crucial for survival. 

**Fig. (1):**
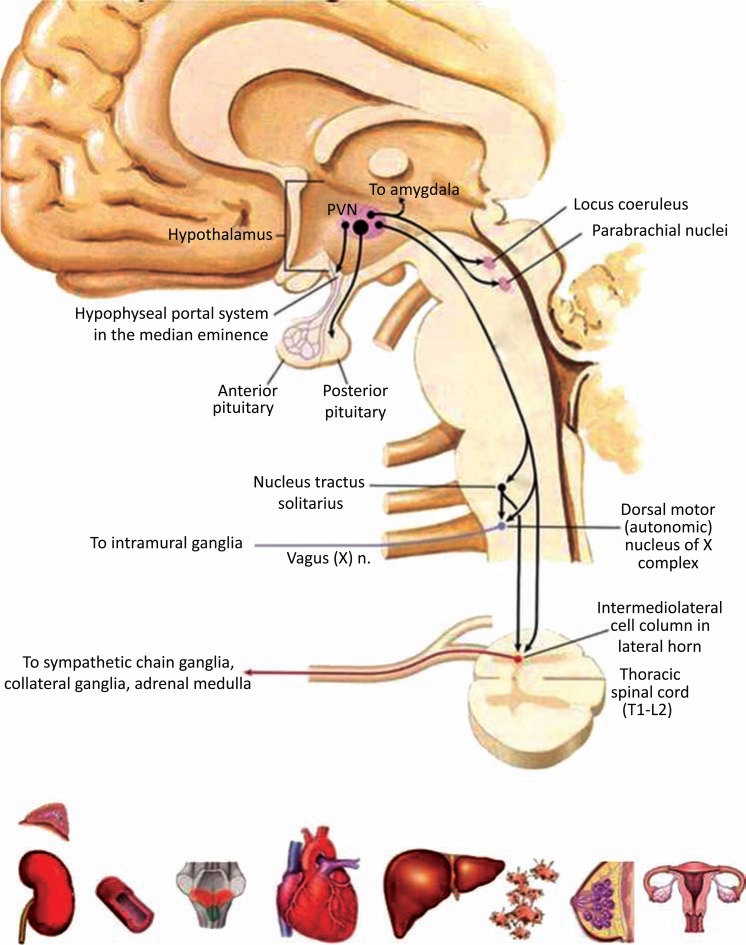
Extra-hypothalamic projections of PVN and targets of neurohypohyseal peptides: kidney, adrenal, blood vessel, area postrema, heart, liver, platelets, breast and uterus [adapted from 189].

Axons of the neurons in the parvocellular part of the PVN also project to extra-hypothalamic areas of the brain, such as the eminentia mediana where VP is released in portal circulation to modulate the release of ACTH during stress response, the limbic system and amygdale where they affect emotions, and the brainstem and intermediolateral column of the spinal cord [2] where VP and OT influence autonomic functions (Fig. **1**). VP and OT released from these axons execute point to point communication participating in *wire* neurotransmission and neuromodulation. The synaptic neurotransmission by peptides, and therefore by VP and OT, have certain characteristics that distinguishes them from classical neurotransmitters. OT and VP released in the synaptic cleft are not subject to degradation and recycling like biogenic amines, they spill over and act extra-synaptically, and the duration of their effects depends on up-and-down-regulation of their receptors [3-5].

VP and OT are also released from the neuronal cell bodies and dendrites into the extracellular space, and this can occur without depolarization. In the extracellular space VP and OT exert autocrine and paracrine regulation and may reduce or enhance the activity i.e. prime magnocellular neurons to engender self-sustained and long-lasting effects. For detailed review refer to [6]. From the extracellular space VP and OT diffuse to the cerebrospinal fluid, use it as vehicle for *volume* communication and reach remote receptor sites, thus overcoming the mismatch between the distribution of OT and VP neurons and their receptors in the brain. 

## STRUCTURE OF VASOPRESSIN, OXYTOCIN AND THEIR RECEPTORS 

Although VP and OT were discovered only in 1953 by Vincent du Vignaud [7, 8] and Roger Acher [9], they appeared some 500 million years ago, in primitive organisms cyclostomes, by duplication of the ancestral gene exposed to irradiation. Their discovery launched the exciting, peptide era in research. VP and OT are almost identical cyclic nonapeptides differing in only two amino acids at position 3 and 8 [10]. Even though subtle, this structural difference between neurohypophyseal peptides imposed considerable diversity in their biological effects. VP and OT are very well known for their roles in osmotic homeostasis and reproduction and a plethora of evidence exists about their contribution to behavior (for excellent review refer to [11]). 

Thanks to the development of pharmacological and genetic tools in research, we now know that VP and OT are involved in the control of many physiological functions in the mammalian as well as in human organism, sometimes in concert and in complementary rather than opposing manner. All the biological effect of OT and VP are mediated through specific, structurally very similar receptors, V1aR, V1bR, V2R and OTR that are widely distributed in the organism, both at the periphery and in the brain [12-19]. The VP and OT receptors have been defined in term of genes, structure [for review refer to 20-22], pharmacology [23-26] and they have been cloned [27-32]. VP and OT receptors are cell membrane receptors with seven trans-membrane domains and are a sub-family within the large super-family of G-protein-linked receptors. Most actions of vasopressin on blood vessel constriction, liver glycogenolysis, platelet adhesion, adrenal angiotensin II secretion, myometrial contractility and brain functions (memory, learning, emotions and autonomic functions) are mediated *via *V1a-type receptors that are coupled to a Gq/11 protein, stimulation of phospholipases C, D and A2 and an increase in intracellular calcium. Vasopressin also stimulates V1bR receptors, discovered long after V1aR receptors, present on pituitary corticotrophs [33] but also in other parts of brain such as the limbic system, in the kidneys and adrenal medulla. Finally, the V2R receptor located in the kidney, mediates water re-absorption in the collecting ducts *via *intracellular cAMP production and translocation of aquaporins in epithelial cell membrane. OT acts *via *OTR receptor, also coupled to a Gq/11 protein, induces myometrial contraction, endometrial prostaglandin F2 alpha production, mammary gland milk ejection, renal natriuresis and specific sexual, affiliative and maternal behaviours [34].

It is important to remember that VP and OT and their receptors are prone to structural changes by mutations and naturally occurring genetic polymorphism that may alter peptide release, receptor distribution and give rise to diverse phenotypes with different predisposition to disease. This topic was tackled by Landgraf and Wigger [35] who identified a single nucleotide polymorphism in the AVP gene promoter region and associated it to over-expression of VP in PVN, dendritic over-release and anxiety/depression trait in rats. Also, genetic variations of V1aR receptor have been associated to pair-bonding behavior both in rodents and humans [36, 37]. Over 150 different genetic mutations of the renal V2R receptor have been described and are associated with the development of diabetes insipidus [38-40]. Only 8 mutations amongst them can be rescued by small molecules (pharmacological chaperones) that penetrate inside the cell and promote the maturation and translocation of the misfolded receptor in the cell membrane [41]. In the following sections, a role of VP and OT in control of the circulation is assessed in a comprehensive manner, both as hormones and as neurotransmitters / modulators.

## HORMONAL (PERIPHERAL) CONTROL OF THE CARDIOVASCULAR SYSTEM BY VASOPRESSIN AND OXYTOCIN

Circulation, along with respiration is the most important vital function. Its primary role is to deliver food (glucose) and oxygen to all tissues and liberate the cell milieu from products of metabolism and CO2. Many factors, neural and endocrine, were found to contribute to the stability of the circulation, and, VP is amongst the key ones. The strongest stimulus to VP release is the increase in plasma osmolality. This information is sensed by the circumventricular organs of the lamina terminalis (subfornical organ, organum vasculosum lamine terminalis) lacking the blood brain barrier and conveyed to PVN [42]. Even subtle changes in VP blood concentration will activate the V2R receptors in the membrane of the principal endothelial cells of the renal collecting duct. Activated V2R receptors increase cAMP and mediate the fusion of intracellular vesicles containing aquaporin 2 water channels with the apical membrane. The increase in number of water pores will engender water re-absorption by diffusion and normalization of blood osmolality with consequent urine concentration [26, 43]. 

VP release can also be engendered by the stimulation of peripheral receptors located in the thoracic vessels that monitor changes in blood pressure (arterial baro-receptors in aorta and carotids, for detailed review refer to [44]), blood volume (receptors at the junction of great thoracic veins to the right atrium, for detailed review refer to [45]) and blood oxygen concentrations (chemo-receptors co-localized with arterial baro-receptors, for detailed review refer to [46]). Information from peripheral receptors travel *via *parasympathetic cranial nerves IX and X to the nucleus of the solitary tract and further to the hypothalamus. VP released in blood, in response to change in blood pressure (BP), targets arterial blood vessels and increases peripheral resistance. A number of *in vitro* and *in vivo* experiments have unequivocally shown that vasopressin is a powerful vasoconstrictor. At molar basis, vasopressin is even more potent than noradrenalin and angiotensin II [47]. However, in some vascular beds vasopressin produces vasodilatation by the stimulation of V2R-like receptors and the release of NO [47-51]. In the preconsticted pulmonary vasculature VP has been found to produce vasodilatation primarily *via *the stimulation of V1 receptors and endothelial release of NO [49, 52].

The effect of OT on blood vessels is less evident. OT is a much weaker constrictor than vasopressin in the systemic circulation *in vitro*, except for the umbilical artery at term [53]. The vasoconstrictor action of OT is mediated *via *V1aR receptors [49, 54]. However, in the presence of increased vascular tone, OT will produce vasodilatation also by the stimulation of V1aR and calcium dependent endothelial NO release [49, 55]. The vasodilatatory effect of OT is evident in the basilary arteries [56]. OT does not seem to play an important physiological role in the regulation of vascular tone in resistance vessels of pregnant rats [57]. 

In a number of studies it was demonstrated that VP administered systemically, peripherally and centrally, in high, nonphysiological i.e. pharmacological doses, increases BP [58, 59] while OT was reported to do the opposite [60]. However, application of selective antagonists, peripherally and centrally, revealed that physiological concentrations of VP and OT do not modulate BP under basal physiological conditions and do not participate in the maintenance of BP under basal physiological conditions [58, 59]. However, V1aR receptor knock-out mice were reported to exhibit lower basal BP values than wild-type controls [61] while OT knock-out mice have been reported to exhibit greater basal BP and heart rate values than wild-type controls [62]. Meticulous investigation of cardiovascular phenotype of V1aR knock-out mice discovered that the hypotension was due to the lack of V1aR receptors in area postrema and consequently blunted baro-receptor reflex control of BP as well as the lack of V1aR receptors in the macula densa of the kidney, which normally facilitates rennin production (lower co-expression of V1aR receptors with nNOS and COX-2 in macula densa diminished synthesis of PGE-2 and NO, potent stimulators of rennin release, [61, 63]). OT receptors were also found in the macula densa of the kidney and were reported to produce natriuresis by the modulation of tuberulo-glomerular feed-back and solute transport [64]. In a series of elegant experiments, Gutkowska and Jankowski, [65] discovered that oxytocin is synthesized in the heart and the vascular smooth muscle and that heart-derived oxytocin regulates atrial natriuretic peptide release and natriuresis in the kidneys [66-68]. It is worth noting that same authors reported that heart-derived OT plays an important role in the heart development and cardiac stem cell renewal and regeneration [65].

## NEUROGENIC CONTROL OF THE CARDIOVASCUALR SYSTEM BY VASOPRESSIN AND OXYTOCIN

A number of studies in the last three decades indicate that VP and OT interfere with feed-back and feed forward mechanisms in neurogenic control of the circulation. 

### Feed-back Mechanisms

VP and OT have been found to be implicated in the modulation of the baro-receptor reflex, stretch-receptor reflex and chemo-reflex. The baro-receptor reflex is the main corrector of blood pressure at beat-to-beat basis, and its malfunction has been associated to bad outcome of cardiovascular disease [69]. The baro receptors are located in aortic arch and carotids and convey information about BP changes *via *vagal afferents to the nucleus of the solitary tract. From NTS, information is further transmitted to other brain areas, primarily to adjacent vagal nuclei and rostral ventrolateral medulla wherefrom baro-receptor efferents arise and set vagal and sympathetic outflow to the heart, and sympathetic outflow to the blood vessels. From the NTS information is also transmitted to the hypothalamus to modulate vasopressin release, diuresis and vascular resistance. VP has been shown to increase BP at plasma concentrations well above anti-diuretic ones, [70] and that this was due to VP-induced decrease in heart rate and renal sympathetic nerve activity (RSNA) initiating renal vasodilatation [71, 72]. Since this effect of VP on HR and RSNA could be prevented by sino-aortic desafferentation it was suggested that VP sensitizes the baro-receptor reflex [73]. Experiments with electrolytic destruction of area postrema, a structure in the fourth cerebral ventricle devoid of blood brain barrier [44, 71, 74], uncovered the site where the sensitization of baro-receptor reflex by VP occurrs. This was further substantiated by identification of abundant neural connections between area postrema and the NTS where baro-receptor afferents terminate [75, 76]. 

The mechanism by which VP acts in area postrema to modulate the baro-receptor reflex remains still unsolved. The majority of work point to V1aR receptors. By intravenous administration of V1aR antagonist, Elliot and coworkers [77] prevented the facilitatory action of VP on baro-receptor reflex, while Michelini and Bonagamba [78], Suzuki and colleagues [79] and Hasser and Bishop [80] provided evidence that microinjection of V1aR antagonist in the area postrema abolished the potentiating effects of vasopressin on renal sympatho-inhibition. However, Brizzee and Walker [72], and Imai and associates [81] found that administration of V2R receptor agonist, desmopressin, to normal rats [72] and Brattleboro rats [81], a strain that lacks endogenous VP, exerts similar effects on baro-receptor reflex as VP, suggesting a role for V2R receptors. Brizee and Walker further reported that the effects of desmopressin (selective V2R agonist) on baro-receptor reflex may be antagonized both by V1aR and V2R selective antagonists. In spontaneously hypertensive rats, and, in rats with renal hypertension, Sampey and co-workers [82] and Nakayama and associates [83] confirmed a role for V2R-like receptors in the regulation of the baro-receptor reflex. Recent cardiovascular phenotypization of V1aR receptor knock-out mice also support a role for V1aR receptors in baro-receptor reflex sensitization [84, 85] but do not rule out the possible role of V2R receptors. Although there is abundance of pharmacological evidence regarding V2R receptors and the modulation of the baro-receptor reflex in the area postrema, there is still no convincing morphological proof for their existence in area postrema [19, 86]. 

While the current opinion is that under basal physiological condition VP does not contribute to the maintenance of BP, a plethora of findings indicates that VP is crucial for maintenance of BP during hemorrhage and that it enhances survival [58, 87-90]. The hypotensive hemorrhage has been documented to be a stimulus to VP transcription and release from PVN and SON [91]. Peripherally released VP produces antidiuresis and vasoconstriction while centrally released VP increases sympathetic outflow to blood vessels and the heart in order to maintain the circulation and thus survival. However, when the blood pressure decreases below the critical value, Peuler and associates [92] provided evidence that VP mediates paradoxical sympatho-inhibition of RSNA and HR. Again, this removal of sympathetic influences to the heart and renal vasculature is crucial for survival because it reduces heart demands for oxygen and preserves the functioning of the kidneys. Fujisawa and coworkers [89], further investigated the mechanism of renal and cardiac sympatho-inhibition using non-peptide V1aR (OPC-21268) and V2R receptor selective antagonist (OPC-31260). They found that V1aR and V2R receptors have opposing effects and that V1aR mediate the inhibition whereas V2R receptors mediate the stimulation of RSNA. In freely moving conscious rats exposed to graded hemorrhaged, using the same selective antagonists, we found that VP modulates BP short-term variability both under nonhypotensive and hypotensive conditions, and that only V2R blockade potentiated hemorrhage-induced bradycardia and prevented the increase of low-frequency BP short-term variability [58] linked to RSNA [93].

Stretch receptors are localized at the junction of great thoracic veins to the right atrium in the so called low pressure high volume compartment of the circulation. Stretch receptor afferents travel *via *the glossopharingeal cranial nerve to the nucleus of the solitary tract and further to the hypothalamus. Stimulation of these receptors produces differential effects on the neural control of the HR and the kidney triggering tachycardia and renal vasodilatation, to accomplish elimination of volume over-load. Since destruction of PVN neurons inhibited renal vascular response to systemic volume load, evidence was provided that PVN is the command centre for low-pressure stretch receptors [94, 95]. Deng and Kaufman [96], further confirmed that upon stimulation of low-pressure receptors at the veno-atrial junction, there is early gene c-fos activation in the parvocellular part of the PVN. In a series of remarkable studies, John Coote and collaborators [97-99] identified two groups of neurons in PVN, one that is activated and the other one that is inhibited by the stimulation of parasympathetic afferents from the right atrium. Also, Strack and coworkers [100] and Schramm and coworkers [101] identified two different pools of neurons in PVN projecting to the spinal cord that influence the heart and the kidney. When Yang and collaborators [102] injected selective V1aR antagonist and OT antagonist at the lower and upper thoracic spinal cord, they dissociated VP and OT neurons in PVN that mediate renal sympatho-inhibition and cardiac sympatho-stimulation, respectively. Altogether these studies revealed that loading stretch receptors at veno-atrial junction engender a unique differential pattern of sympathetic activity to the heart and the kidney *via *PVN direct projections to the intermediolateral column of the spinal cord: OTR receptors were found to mediate cardiac sympathetic activation and tachycardia while V1aR receptors were found to produce renal symaptho-inhibition, with consequent renal vasodilatation and diuresis. For detailed review refer to [45].

Chemo-receptor reflex is another important regulation mechanism in determination of neurogenic control of the circulation. Chemo receptors are co-localized with baro-receptors and use the same parasympathetic afferent to the NTS to convey information to the brain on blood oxygenation. Early as 1963 Redgate [103] first noticed that hypothalamic lesion depressed ventilation but did not go further in elaborating the mechanism. Almost half a century later Duan and coworkers [104] and Schlenker and collaborators [105] have shown in conscious and anesthetized animals, that PVN alters cardio-respiratory function in parallel. Functional and anatomical studies have indicated that the stimulus to sympatho-respiratory excitation is hypoxia, not hypercapnia, and that the information travels *via *the carotid sinus nerve and activates the commissural nuclei of the solitary tract that further project to PVN [106-108]. Only when Yeh and coworkers [109] identified direct connections between the PVN and the phrenic motoneurons, as well as indirect connection of PVN with brainstem bulbospinal neurons, that morphological basis for the neuronal path for concomitant cardio-respiratory control was provided. Kc and coworkers [110] and Mack and coworkers [111], further investigated the mechanisms involved in this neuronal communication. They discovered that both VP and OT neurons from the parvocellular part of the PVN project to the pre-Bötzinger complex and phrenic motoneurons, and that only VP-containing neurons project to rostral ventrolateral medulla, a vasomotor centre [46, 112]. They also provided evidence on the abundance of V1aR and OTR receptors in the rostral ventral respiratory column including the pre-Bötzinger complex and rostral ventrolateral medulla. Altogether these experiments revealed that PVN stimulation increased neuronal discharge of VP and OT containing neurons to the pre-Bötzinger complex and rostral ventorlateral medulla. Consequently, BP and HR increased with respiration [46, 110, 112, 113]. Using the advantages of spectral analysis technique that provides a dynamic insight into cardio-respiratory control (for review refer to [114]) Japundžić-Žigon and coworkers provided equivalent evidence in conscious rats, that endogenously released VP during hemorrhage [58, 115] or stress [59] concomitantly increased sympathetic outflow to blood vessels and the depth of respiration. They also showed that centrally injected cholinergic drugs produced concomitant increase of respiration-induced high-frequency variability and sympathetically mediated low-frequency short-term variability of BP involving central vasopressinergic pathways and V1aR receptors [116].

### Feed Forward Mechanisms

The feed-forward command or positive feedback control of the circulation is activated during stress and exercise and opposes negative feed-back influences. In 1980, Ciriello and Calaresu [117] first reported that stimulation of SON and PVN inhibited reflex bradycardia elicited by carotid sinus nerve stimulation, and, Matsuguchi and coworkers [118] reported that microinjections of VP in nucleus of the solitary tract elicited concomitantly hypertension and tachycardia. Furthermore Schmid and collaborators [119], showed that intracerebroventricularly injected VP as well as micro-injections of VP and OT in nucleus of the solitary tract, increased sympathetic vasomotor drive and blunted the baro-receptor reflex. These finding were surprising and in collision with previous data about the sensitization of the baro-receptor reflex by VP in area postrema. Then, Unger and coworkers [120] provided evidence that peripherally applied VP sensitized the baro-receptor reflex by the stimulation of V2R receptors in area postrema, while centrally applied VP inhibited the baro-receptor reflex by the stimulation of central V1R receptors, inaccessible from blood. Michelini and Bonagamba [78], further demonstrated that increased concentration of VP in the nucleus of the solitary tract attenuated baro-receptor reflex sensitivity, and suggested that neuronal projections from PVN might be involved in exercise-induced increases of BP [121, 122] Moreover, Morris and collaborators [123] found that OT containing neurons in PVN mediate stress-induced tachycardia. It was also shown that centrally injected VP attenuated reflex bardycardia and that VP antagonists enhanced it [124]. Li and collaborators [125, 126] elaborated the mechanisms in PVN. According to them, GABA tonically inhibits glutamate excitatory inputs to pre-sympathetic neurons in PVN. Excitatory glutamate inputs trigger NO release that act as facilitator of GABA, closing the negative feed-back loop. We have tackled another mechanism. We performed microinjections of adenoviral vector to transfect the PVN magnocellular neurons of rats with naturally occurring V1aR receptors [127], and we noted that rats over-expressing V1aR receptors in PVN under basal physiological conditions have reduced baro-receptor reflex sensitivity. These finding open another possibility that increased expression of vasopressin V1aR receptors in PVN might trigger dendritic release of VP, induce priming of PVN neurons and blunt the reflex (for review refer to [127]). 

## VASOPRESSIN, OXYTOCIN AND CARDIOVASCULAR RESPONSE TO STRESS

It is widely acknowledged that chronic psychological stress is the risk factor in etiopathogenesis of cardiovascular disease, and that acute stress can trigger cardiac events such as myocardial infarction and sudden death [128]. During exposure to stress the cardiovascular response is driven by the emotional and the behavioral reaction. Generally there are two different behavioural strategies, the active coping strategy and the passive coping strategy, each of which is associated to differential neurogenic cardiovascular response [129, 130]. It is well established that vasopressin is important for the normal response to stress both as the modulator of the hypothalamo-pituitary [131, 132] and the sympatho-adrenal axes [133]. The pattern of involvement of VP in the stress response depends on the type of stressor. For instance Stojičić and coworkers [134, 135] demonstrated that in rats exposed to air-jet stress, a model that induces fear and panic reaction with escape (active coping strategy), VP mediates the neurocardiogenic response but do not affect the endocrine response as measured by blood corticosterone level before and after exposure to stress. Also, accumulated experimental evidence indicates that the modulation of hypothalamo-pituitary axes by VP is important for adaptation to stress [136, 137], suggesting that impairment of habituation mechanisms may also be important for developing pathology. For instance borderline hypertensive rats exposed to repeated stress in the pre-hypertensive stage, exhibit impaired neurogenic cardiovascular control and allostatic overload [138].

Using the advantages of spectral analysis technique that provide a dynamic insight into cardiorespiratory control [114], Milutinović and coworkers [59] have shown that, endogenous increase of VP concentration in the brain during exposure of rats to stress by immobilization, enhances the respiration induced high-frequency blood pressure variability and sympathetically-mediate LF-BP variability directed to blood vessels [139, 140]. This effect of VP may be crucial for survival because increasing the depth of respiration has dual beneficial effect for maintaining circulation: to increase the available alveolar surface for most efficient blood oxygenation and to assist in heart filling by greater aspiration of blood in inspiration (negative intra-thoracic pressure). The results of Milutinović and coworkers [59] also indicate that during stress induced by immobilization, there is a V1aR receptor-mediated increase in respiratory sinus arrhythmia or high-frequency oscillation of heart rate, a well known, physiological vago-vagal phenomenon. This could be also important in protecting the heart from sympathetic over-stimulation during stress. Interestingly we noticed that both central V1aR and V1bR receptor blockade abolished stress-induced increase of respiration derived blood pressure short-term variability. The possibility that V1aR receptors in the preBötzinger complex and rostral ventrolateral medulla could mediate this effect, was substantiated by morphological and functional findings by Kc and colleagues [46, 110, 112]. Our results also suggest that VP may control respiration from more than one central site. There is another possibility that blockade of V1bR receptors in the bed nucleus of the stria terminalis that projects to PVN and exerts anxiolytic/antidepressant effect [141-143], alleviates the emotional response to stress and pacifies respiration. The upper panel of Fig. (**2**) illustrates the increase of blood pressure and blood pressure low-frequency and high-frequency short-term variability in one rat during exposure to emotional stress, due to simultaneous increase of sympathetic outflow to resistance vessels and respiration, respectively. Lower panel demonstrates how pre-treatment of rats with V1b antagonist abolished the effects of stress on both sympathetically- and respiration-mediated blood pressure variability. Since the V1b antagonist was injected previous to exposure to stress in the lateral ventricle of the rat, it is reasonable to propose that V1b antagonist acted at the neighboring brain structure with abundance of V1b receptors such as bed nucleus of the stria terminalis, well recognized to be involved in the emotional response to stress [143]. Recently, Lolait and collaborators [144-146] reported that V1bR knock-out mice, as well as mice pre-treated with V1bR receptor antagonist, exhibit diminished neuro-endocrine response to stress, as measured by the release of ACTH and blood corticosterone response, complementing our findings.

A number of animal studies suggest that affiliative social interactions elicit an increase in OT activity which then activates an anti-stress response that promotes bonding, relaxation and growth, while reducing cardiovascular and neuroendocrine stress responsivity [147-152]. For instance OT is found to blunt restraint-induced hypothalamo-pituitary axes activation [150, 151], to decrease cardiovascular responding to isolation [147], to reduce anxiety-like behavior [150] and promote social interactions [149]. Krause and collaborators [148] also found that OT mediates a buffering effect of hypernatremia on stress-induced behavioural, endocrine and cardiovascular response. In OT knock-out mice, Bernatova and co-workers [62] described accentuated BP and corticosterone response during exposure to acute stress. In line with their finding Wsol and coworkers [152] reported that central application of OTR receptor antagonist enhanced BP and HR increase to environmental stress. Clinical findings also support a role for OT as an anti-stress hormone. Altemus and co-workers [53] reported that lactating women have greater parasympathetic control of the heart, and Grewen and Light [153] found that plasma OT in lactating women is related with lower cardiovascular reactivity to stress. 

It is noteworthy mentioning that pregnancy is a physiological state where important cardiovascular hemo-dynamic changes occur to meet the oxygen and nutritional requirements of the growing uterus and developing fetus. Strong evidence exists that VP and OT are involved in the mediation of the hypervolaemic and hyponatremic state in pregnancy (for detailed review see [154]). By the end of pregnancy blood volume is increased by 55% and plasma sodium concentration and osmolality are decreased by 4% [154], and this is induced by corpora lutea derived relaxin that stimulates VP release and drinking behavior [155]. At the same time OT neurons remain quiescent [156]. The mechanism of OT neurons quiescence to osmotic stimuli in pregnancy remains unexplored though allepregnenolon, a progesterone neurosteroid metabolite, was implicated [157-159]. As a consequence of hypervolaemia, maternal cardiac output increases and this is accompanied by gradual increase of myocardial contractility provoking mild left ventricular hypertrophy by the end of pregnancy [160]. At the same time remodeling of cardiovascular neurogenic control occurs and the baro-receptor reflex sensitivity is decreased (for review refer to [161]), as well as the peripheral resistance and blood pressure. Moreover, the responsiveness of maternal vessels to pressor agents is reduced while the responsiveness to vasodilators is enhanced. At this point there is no evidence that VP and OT contribute to neurogenic and hemodynamic remodeling in pregnancy [57, 162]. 

## PATHOPHYSIOLOGICAL IMPLICATIONS 

Several lines of experimental evidence suggest that VP contributes to the pathogenesis of hypertension. In genetically hypertensive rats [115, 163-168] and rats with endocrine hypertension [169] V1aR receptor antagonists have been found to reduce BP. Moreover, spontaneously hypertensive rats and humans have been shown to have elevated plasma VP concentration [170] and exhibit enhanced vascular responsiveness to exogenously applied VP [82, 171]. However this elevation of VP in blood of hypertensive animals and humans is insufficient to justify the increase of BP in hypertension; it rather correlates well to the severity of hypertension [170]. Some earlier reports have suggested that chronic stimulation of V1R receptors in normotensive rat resulted in sustained hypertension [172, 173]. A more recent work by the same author [174] demonstrated that VP, even in sub-pressor doses, produced sustained hypertension in Dahl salt sensitive rats with impaired renal medullary NO that mediates V2R receptor vasodilatation. Moreover, in spontaneously hypertensive rats, deoxicorticosterone acetate-salt hypertension, rennin transgenic hypertension and reno-vascular hypertension, there is an over-activation of the brain vasopressinergic system and altered expression of V1R receptors in the brain [175-177]. Yi and collaborators [178] have also reported increased VP synthesis in PVN and SON of spontaneously hypertensive rats that develop stroke. Petersson and coworkers [179] investigated the role of OT in hypertension. They reported that OT has an antihypertensive effect in spontaneously hypertensive male rats. We have noted that, in spontaneously hypertensive rats a buffering effect VP on BP short-term variability is impaired [115], and postulated that this failure could contribute to increased BP variability, an independent risk marker for end-organ damage in hypertension [180]. Altogether, experimental and clinical data suggest that VP does not play a key role in the etiology of hypertension, but that it rather impinges on the severity of disease and its prognosis.

It is now well established that VP contributes to the pathogenesis of heart failure. Enhanced release of VP in heart failure is initiated by the decrease of cardiac output and subsequent stimulation of the baro-receptor reflex. VP released in the circulation acts to increase blood volume and arterial resistance, while centrally liberated vasopressin increases sympathetic outflow to the cardiovascular system [181], burdening the failing heart and rising the risk for arrhythmias and sudden death. Increased sympathetic drive, circulating angiotensin II and tumor necrosis factor α (released from the failing heart) also stimulate VP release, closing the positive feed-back loop [133, 182-184]. In the PVN of animals with failing heart a reduction of GABA content was reported suggesting removal of the tonic inhibition of pre-sympathetic neurons [185, 186]. At present, V2R receptor antagonists – vaptans that act peripherally to produce water diuresis, have been introduced in the treatment of hyponatremic hypervolaemia associated with advanced stages of heart failure. Unfortunately vaptans do not appear to delay the progression of heart failure or to decrease mortality [187]. For novel central mechanisms and challenges for new drug development in heart failure refer to [188]. 

## CONCLUSION AND PERSPECTIVES

There is no doubt that neurohypophyseal peptides VP and OT, both as hormones and neurotransmitters/neuromodulators influence vital functions, the cardiovascular system and respiration. The modulation of the cardiovascular system occurs at many levels, the kidneys, the vasculature, and the nervous system. In the kidneys, VP and OT act in complementary manner to maintain osmotic homeostasis: while VP conserves water OT eliminates sodium. Remodeling of VP and OT neurons is crucial in osmotic adaptation to pregnancy. In blood vessels, VP and OT are both vasoconstrictors and increase peripheral resistance, but they can also produce vasodilatation in the lungs and the brain. In the brain, VP and OT act like enhancers of sympathetic outflow and respiration, while peripherally, VP augments the sensitivity of the baro-receptor reflex *via *the area postrema. Therefore VP and OT are important in the cardiovascular response to stress. While VP supports the activation of the hypothalamo-pituitary and sympatho-adrenal axes, OT buffers the effects of stress on the cardiovascular system. Evidence has been provided that vasopressinergic mechanisms contribute to deterioration of hypertension and heart failure and that OT has hypotensive properties and promotes cardiac regeneration. However we still know very little about the process within the PVN that dictates neurocardiogenic remodeling in cardiovascular disease. We also need to elucidate the role of OT in cardiovascular pathology and evaluate its potential in cardiovascular healing. 

## Figures and Tables

**Fig. (2):**
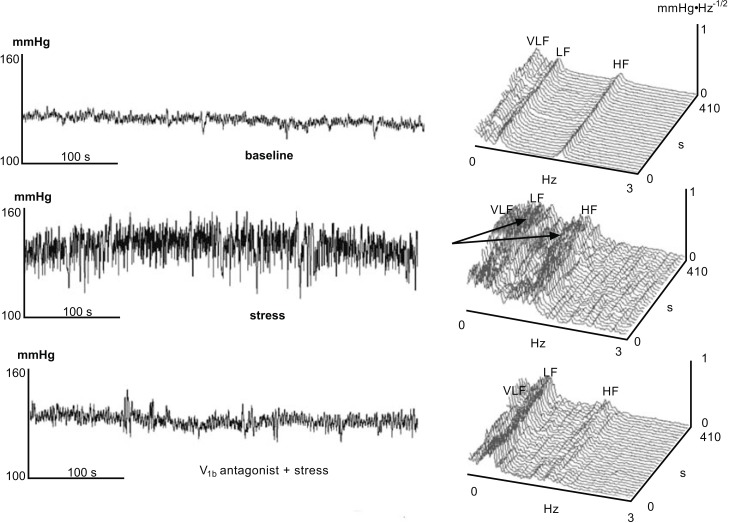
A 7 minutes-long recording of systolic arterial pressure (BP) in adult male Wistar rat and the corresponding time spectrum under basaline conditions (upper panel), during exposure to stress by immobilization without V1bR receptor antagonist pre-treatment (middle panel) or with V1bR receptor antagonist pre-treatment (lower panel). Arrows indicate simultaneous increase of the respiratory, high frequency (HF) and sympathetic low frequency (LF) oscillations of systolic BP induced by acute immobilization [59].

## ABBREVIATONS

VP: = Vasopressin
OT: = Oxytocin
V1aR: = vasopressin V1a receptor
V1bR: = vasopressin V1b receptor
V2R: = vasopressin V2 receptor
OTR: = oxytocin receptor
SON: = supraoptic nucleus
PVN: = paraventricular nucleus
ACTH: = adrenocorticotrophic hormone
BP: = blood pressure
HR: = heart rate
LF: = low-frequency
HF: = high-frequency
nNOS: = neural nitric oxide synthase
COX-2: = cyclooxigenase – 2
NO: = nitric oxide
PGE2: = prostaglndin E2
GABA: = gamma aminobutiryc acid
